# Supplementary material for: BTK Isoforms p80 and p65 Are Expressed in Head and Neck Squamous Cell Carcinoma (HNSCC) and Involved in Tumor Progression
Source: Cancers (Basel). 2023 Jan 3;15(1):310. doi: 10.3390/cancers15010310 (PMC9818583; doi:10.3390/cancers15010310)
Supplement: Supplementary file 1 [file cancers-15-00310-s001.zip › cancers-2070123-supplementary.pdf]

Supplementary Material

BTK Isoforms p80 and p65 Are Expressed in Head and Neck Squamous Cell Carcinoma (HNSCC) and Involved in Tumor Progression

Annika C. Betzler [1], Hannah Strobel [1], Tsima Abou Kors [1], Jasmin Ezić [1], Kristina Lesakova [1], Ronja Pscheid [1], Ninel Azoitei [2], Johanna Sporleder [1], Anna-Rebekka Staufenberg [1], Robert Drees [1], Stephanie E. Weissinger [3], Jens Greve [1], Johannes Doescher [1], Marie-Nicole Theodoraki [1], Patrick J. Schuler [1], Simon Laban [1], Toshiro Kibe [4], Michiko Kishida [4], Shosei Kishida [4], Christian Idel [5], Thomas K. Hoffmann [1], Marialuisa Lavitrano [6], Emanuela Grassilli [6] and Cornelia Brunner [1,*]

[1] Department of Oto-Rhino-Laryngology, Ulm University Medical Center, 89075, Ulm, Germany;
[2] Department of Internal Medicine I, Ulm University Medical Center, 89081, Ulm, Germany;
[3] Institute of Pathology, Ulm University Medical Center, 89081, Ulm, Germany;
[4] Department of Biochemistry and Genetics, Graduate School of Medical and Dental Sciences, Kagoshima University, 890-8580, Kagoshima, Japan;
[5] Department of Otorhinolaryngology, University of Luebeck and University Hospital Schleswig-Holstein, Campus Luebeck, 23538, Luebeck Germany
[6] School of Medicine and Surgery, University of Milano-Bicocca, 20900, Monza, Italy;

* Correspondence: cornelia.brunner@uniklinik-ulm.de

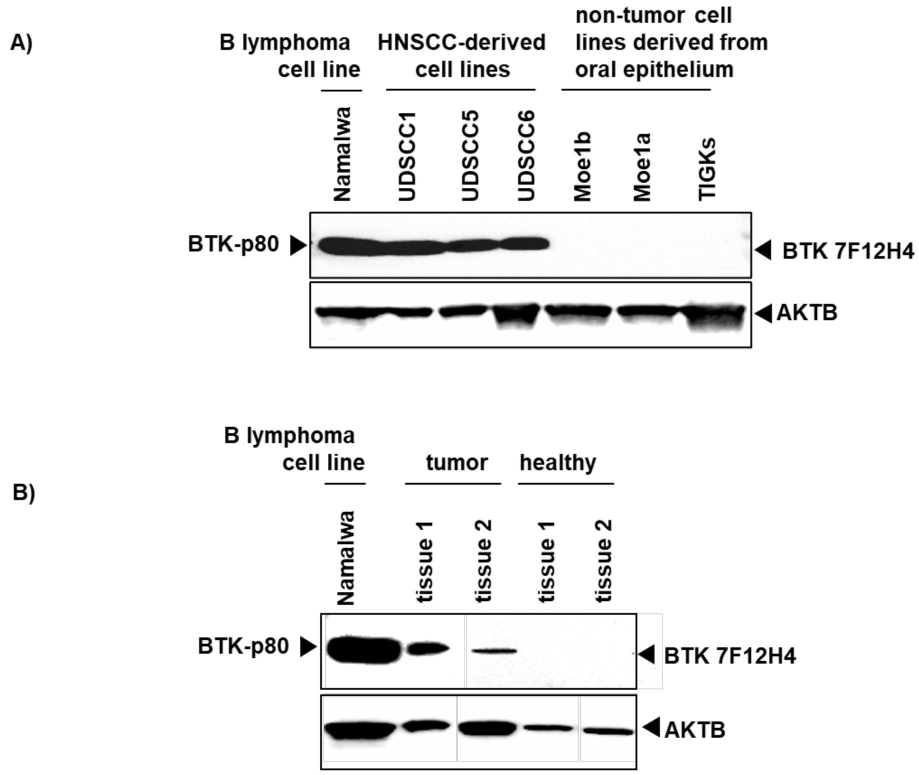

<u>Figure S1:</u> (**A**) Expression of BTK-p80 isoform was determined in one B cell lymphoma cell line (Namalwa), three HNSCC-derived cell lines (UDSCC1, UDSCC5, UDSCC6) as well as in non-tumor cell lines derived from oral epithelium (Moe1a, Moe1b, TIGK) by immunoblotting using BTK antibody 7F12H4. (**B**) Expression of BTK-p80 was determined in primary cancer tissue specimens of the head and neck region and in healthy non-tumor squamous epithelial cells obtained from the same individuals using BTK antibody 7F12H4. β-actin was used as loading control.

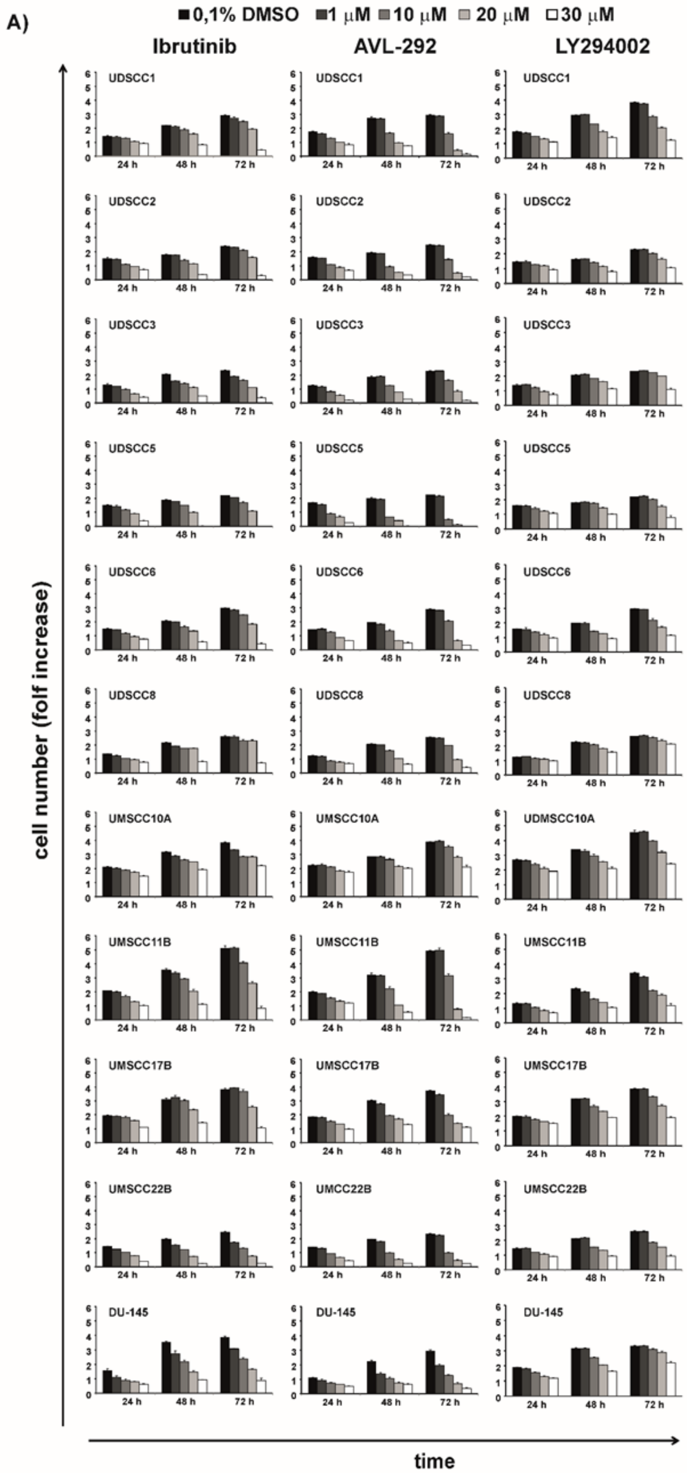
A)
0,1% DMSO 1 μM 10 μM 20 μM 30 μM
Ibrutinib
AVL-292
LY294002
UDSCC1
UDSCC2
UDSCC3
UDSCC5
UDSCC6
UDSCC8
UMSCC10A
UDMSCC10A
UMSCC11B
UMSCC17B
UMSCC22B
UMCC22B
DU-145
24 h
48 h
72 h
cell number (folf increase)
time

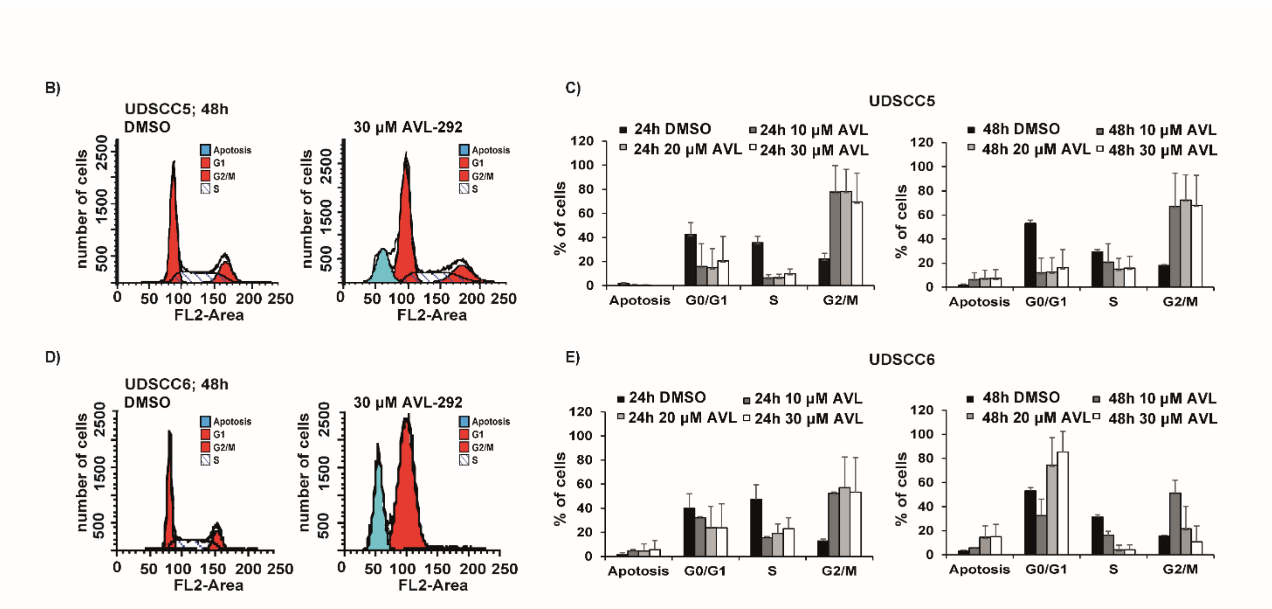

Figure S2: BTK inhibition impairs proliferation and induces cell cycle arrest and apoptosis. (**A**) BTK inhibition decreased proliferation of HNSCC-derived cell lines. Ten HNSCC-derived cell lines and the prostate carcinoma cell line DU-145 were seeded and treated with different concentrations (1, 10, 20 and 30 µM) of three different BTK inhibitors (Ibrutinib, AVL-292 and LY294002). Treatment with 0.1 % DMSO served as control. Proliferation was assessed over a time. This experiment was performed in triplicates. Presented are the mean values. (**B-E**) BTK inhibition induces cell cycle arrest and apoptosis. Cell cycle and apoptosis were analyzed using UDSCC5 (**B, C**) or UDSCC6 (**D, E**) cells that were either left untreated (DMSO control) or treated with different concentrations (10, 20 or 30 µM) of AVL-292. Cell cycle and apoptosis was assessed 24h and 48h after treatment. Representative graphs of cell cycle analyses are shown in D and F. Depicted in E and G are mean values ± SD of three independent experiments.

Original blots of Figure 1F

<table>
<tr>
<td>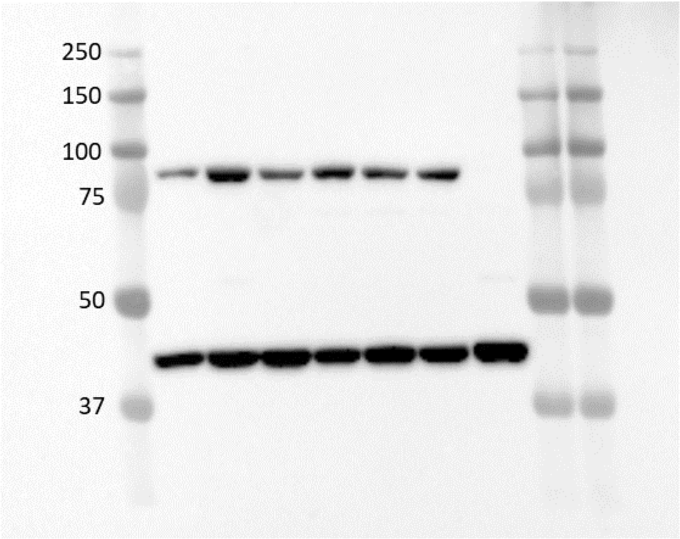
</td>
<td>BTK 7F12H4
(80 kDa)

ß-actin
(42 kDa)</td>
<td rowspan="3">Lane 1: Marker
Lane 2: UD-SCC1
Lane 3: UD-SCC5
Lane 4: UD-SCC6
Lane 5: Ramos
Lane 6: Namalwa
Lane 7: Preb1
Lane 8: CD19+ cells
Lane 9: Marker
Lane 10: Marker</td>
</tr>
<tr>
<td>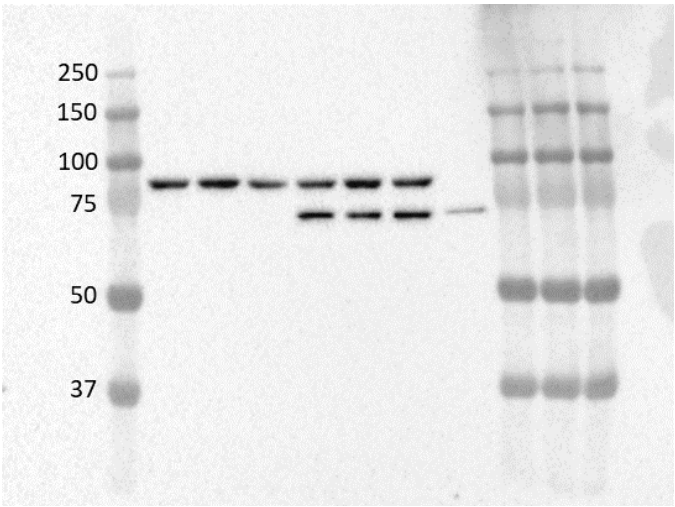
</td>
<td>BTK DFS
(80 and 77 kDa)</td>
</tr>
<tr>
<td>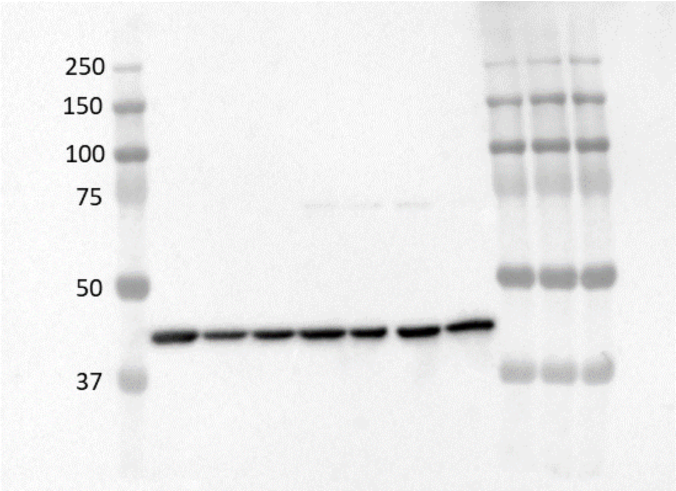
</td>
<td>ß-actin
(42 kDa)</td>
</tr>
</table>

Original blots of Figure 1G (right panel)

<table>
<tr>
<td>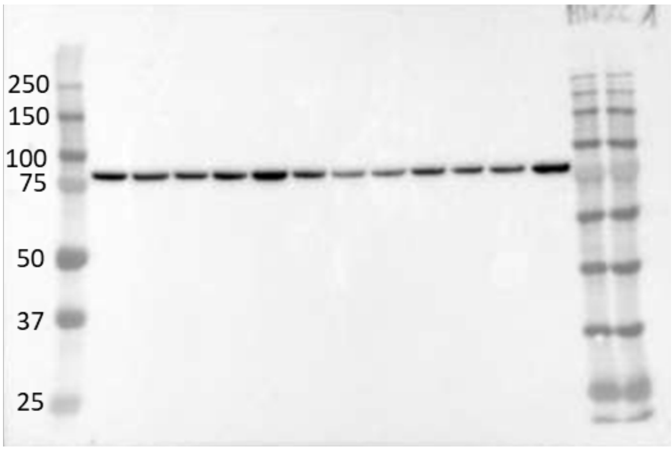
</td>
<td>BTK 7F12H4
(80 kDa)</td>
<td rowspan="3">Lane 1: Marker
Lane 2: Namalwa
Lane 3: UD-SCC1
Lane 4: UD-SCC2
Lane 5: UD-SCC3
Lane 6: UD-SCC5
Lane 7: UD-SCC6
Lane 8: UD-SCC8
Lane 9: UD-SCC10A
Lane 10: UM-SCC11B
Lane 11:UM-SCC17B
Lane 12:UM-SCC22B
Lane 13: Marker
Lane 14: Marker</td>
</tr>
<tr>
<td>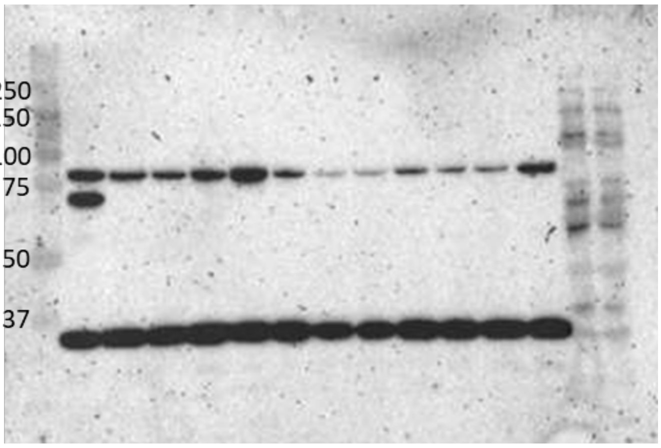
</td>
<td>BTK DFS
(80 and 77 kDa)</td>
</tr>
<tr>
<td>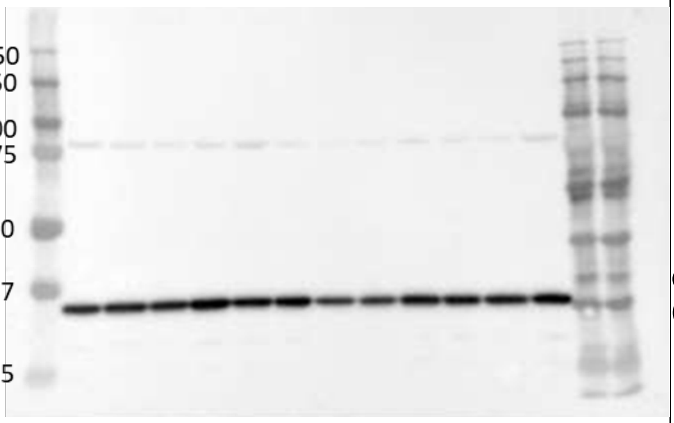
</td>
<td>GAPDH
(37 kDa)</td>
</tr>
</table>

Original blots of Figure 1G (left panel)

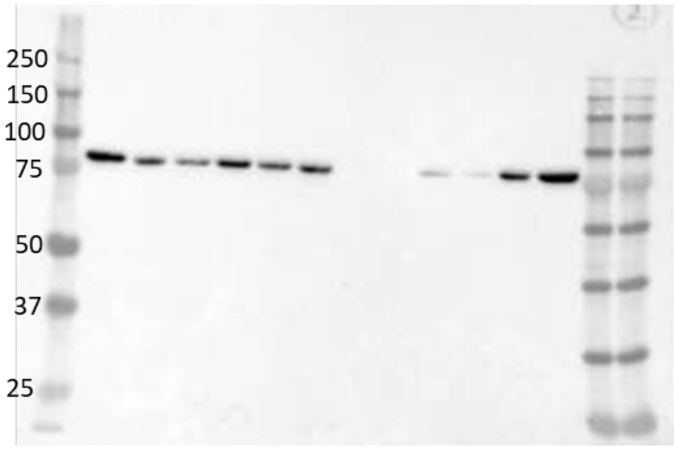	**BTK 7F12H4**<br>(80 kDa)	Lane 1: Marker<br>Lane 2: Namalwa<br>Lane 3: not of interest<br>Lane 4: not of interest<br>Lane 5: DU-145<br>Lane 6: LNCaP<br>Lane 7: PC-3<br>Lane 8: not of interest<br>Lane 9: not of interest<br>Lane 10: not of interest<br>Lane 11: not of interest<br>Lane 12: not of interest<br>Lane 13: Marker<br>Lane 14: Marker
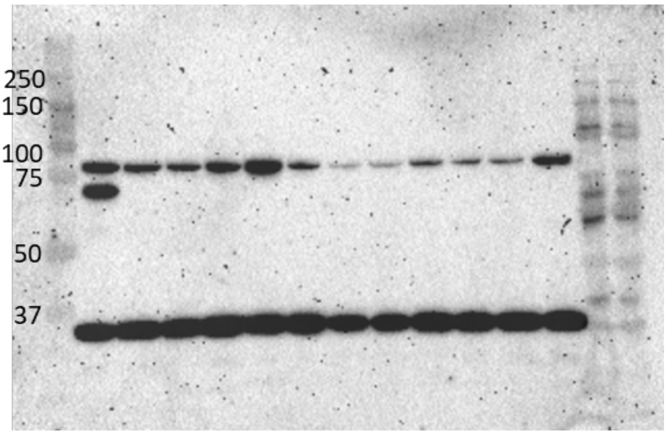	**BTK DFS**<br>(80 and 77 kDa)	
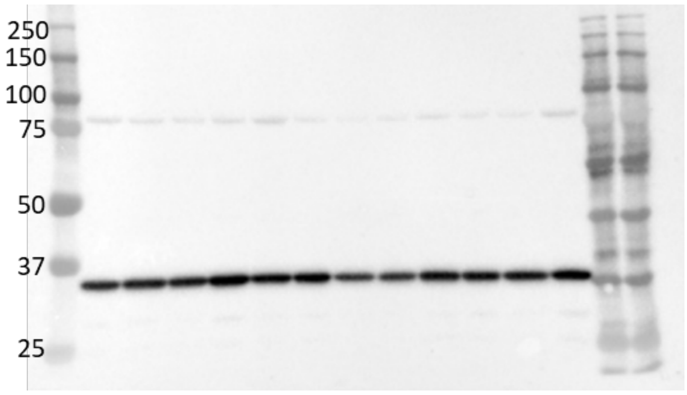	**GAPDH**<br>(37 kDa)	

Original blots of Figure 1H

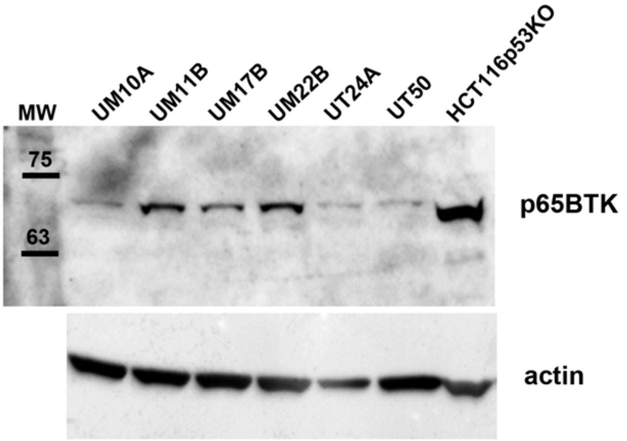

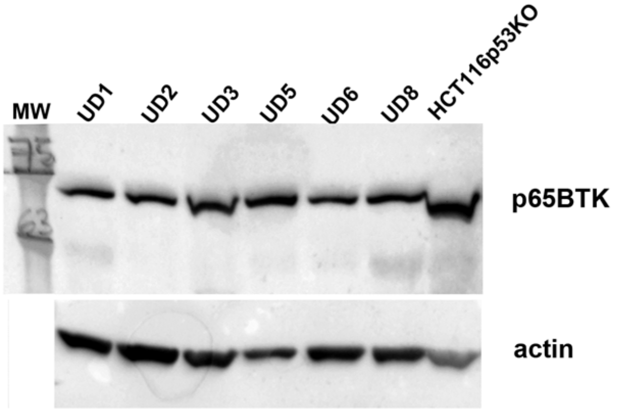

<u>Figure S3:</u> Original membranes of immunoblots shown in Figure 1 F-H.

Original blots of Figure 3

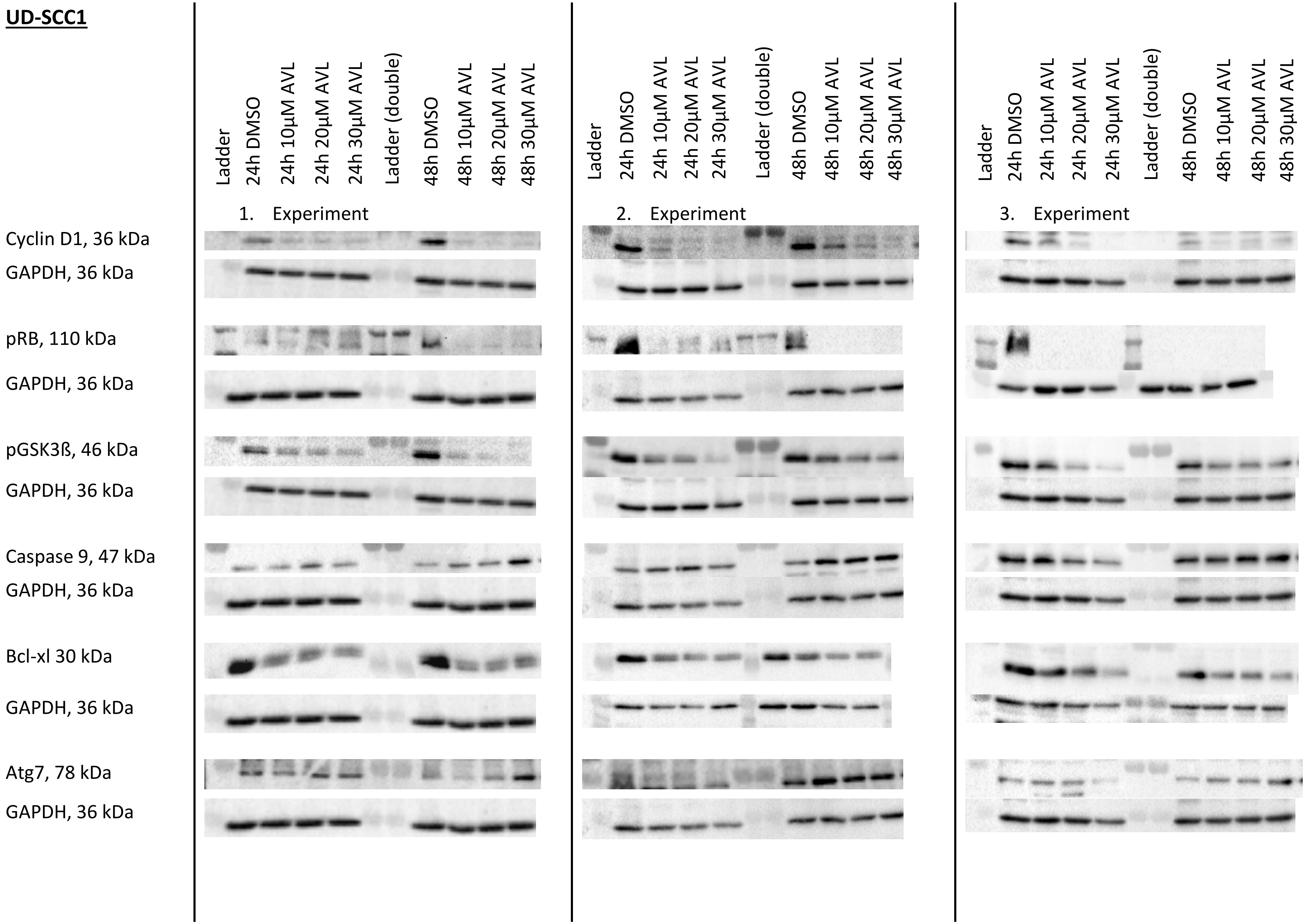

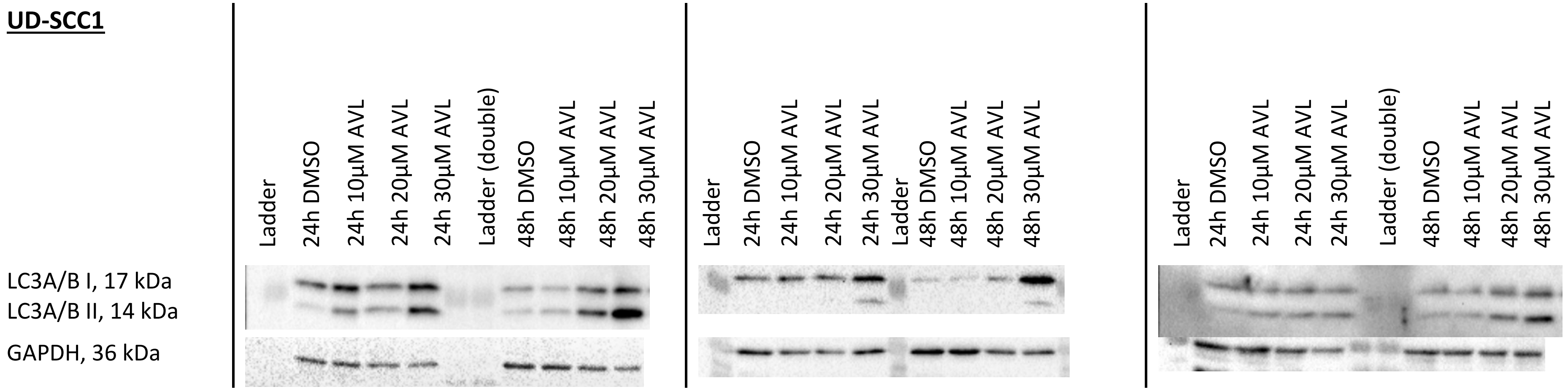
UD-SCC1
Ladder
24h DMSO
24h 10µM AVL
24h 20µM AVL
24h 30µM AVL
Ladder (double)
48h DMSO
48h 10µM AVL
48h 20µM AVL
48h 30µM AVL
Ladder
24h DMSO
24h 10µM AVL
24h 20µM AVL
24h 30µM AVL
Ladder
48h DMSO
48h 10µM AVL
48h 20µM AVL
48h 30µM AVL
Ladder
24h DMSO
24h 10µM AVL
24h 20µM AVL
24h 30µM AVL
Ladder (double)
48h DMSO
48h 10µM AVL
48h 20µM AVL
48h 30µM AVL
LC3A/B I, 17 kDa
LC3A/B II, 14 kDa
GAPDH, 36 kDa

UD-SCC 2

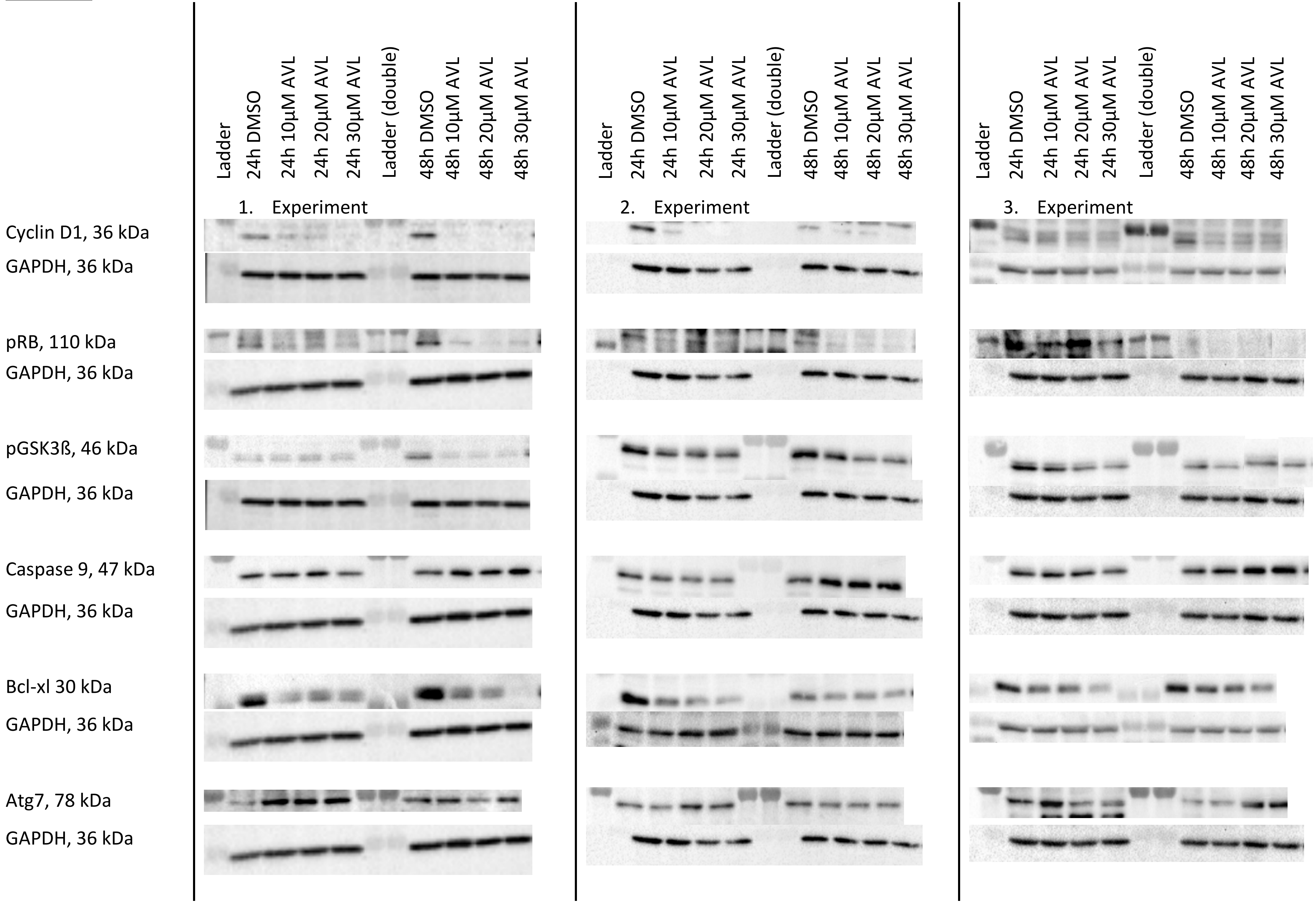
Ladder
24h DMSO
24h 10µM AVL
24h 20µM AVL
24h 30µM AVL
Ladder (double)
48h DMSO
48h 10µM AVL
48h 20µM AVL
48h 30µM AVL
1. Experiment
2. Experiment
3. Experiment
Cyclin D1, 36 kDa
GAPDH, 36 kDa
pRB, 110 kDa
GAPDH, 36 kDa
pGSK3ß, 46 kDa
GAPDH, 36 kDa
Caspase 9, 47 kDa
GAPDH, 36 kDa
Bcl-xl 30 kDa
GAPDH, 36 kDa
Atg7, 78 kDa
GAPDH, 36 kDa

UD-SCC 2

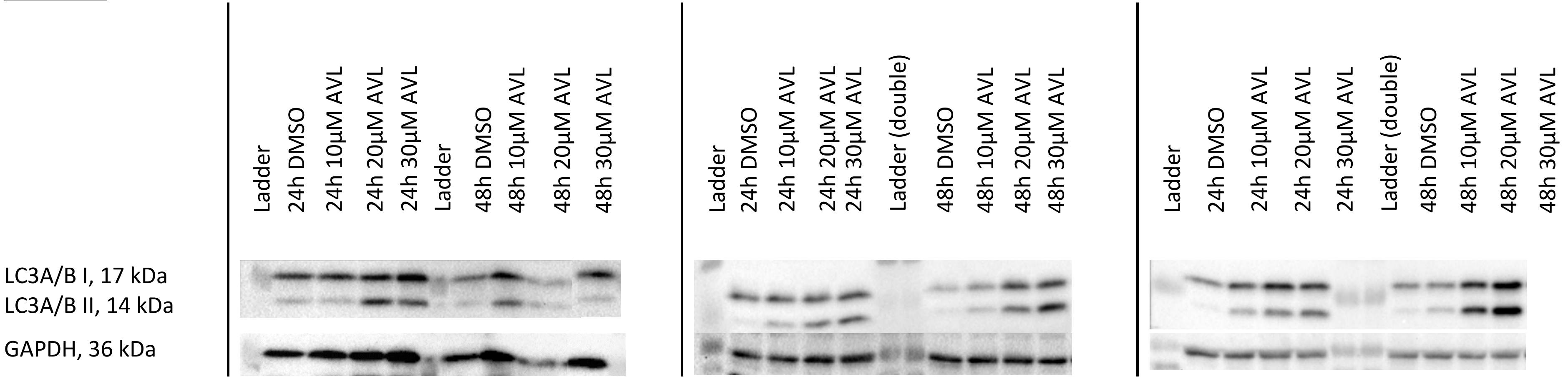

LC3A/B I, 17 kDa
LC3A/B II, 14 kDa
GAPDH, 36 kDa
Ladder
24h DMSO
24h 10µM AVL
24h 20µM AVL
24h 30µM AVL
Ladder
48h DMSO
48h 10µM AVL
48h 20µM AVL
48h 30µM AVL
Ladder
24h DMSO
24h 10µM AVL
24h 20µM AVL
24h 30µM AVL
Ladder (double)
48h DMSO
48h 10µM AVL
48h 20µM AVL
48h 30µM AVL
Ladder
24h DMSO
24h 10µM AVL
24h 20µM AVL
24h 30µM AVL
Ladder (double)
48h DMSO
48h 10µM AVL
48h 20µM AVL
48h 30µM AVL

UD-SCC 6

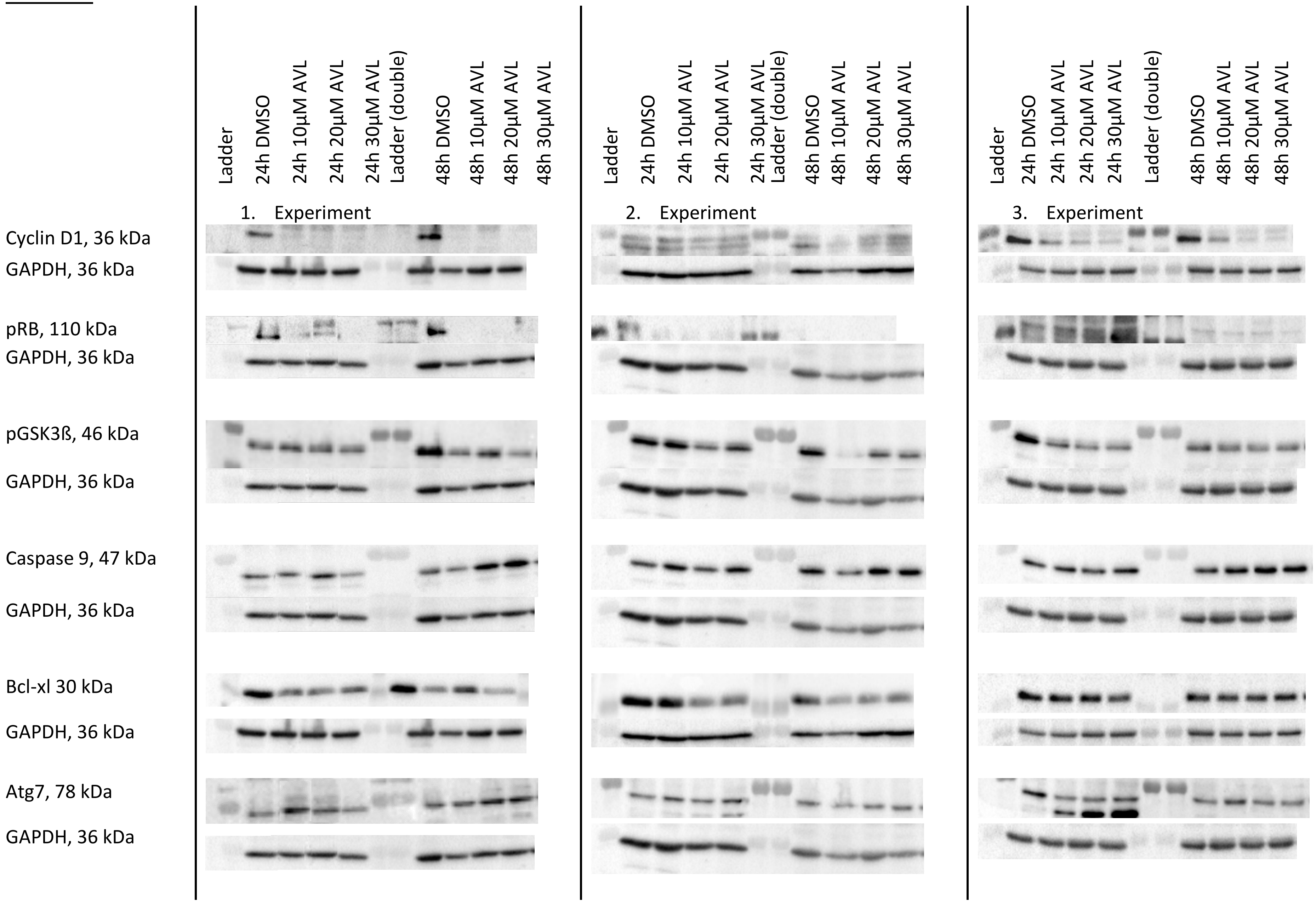
Ladder
24h DMSO
24h 10µM AVL
24h 20µM AVL
24h 30µM AVL
Ladder (double)
48h DMSO
48h 10µM AVL
48h 20µM AVL
48h 30µM AVL
1. Experiment
2. Experiment
3. Experiment
Cyclin D1, 36 kDa
GAPDH, 36 kDa
pRB, 110 kDa
GAPDH, 36 kDa
pGSK3ß, 46 kDa
GAPDH, 36 kDa
Caspase 9, 47 kDa
GAPDH, 36 kDa
Bcl-xl 30 kDa
GAPDH, 36 kDa
Atg7, 78 kDa
GAPDH, 36 kDa

UD-SCC 6

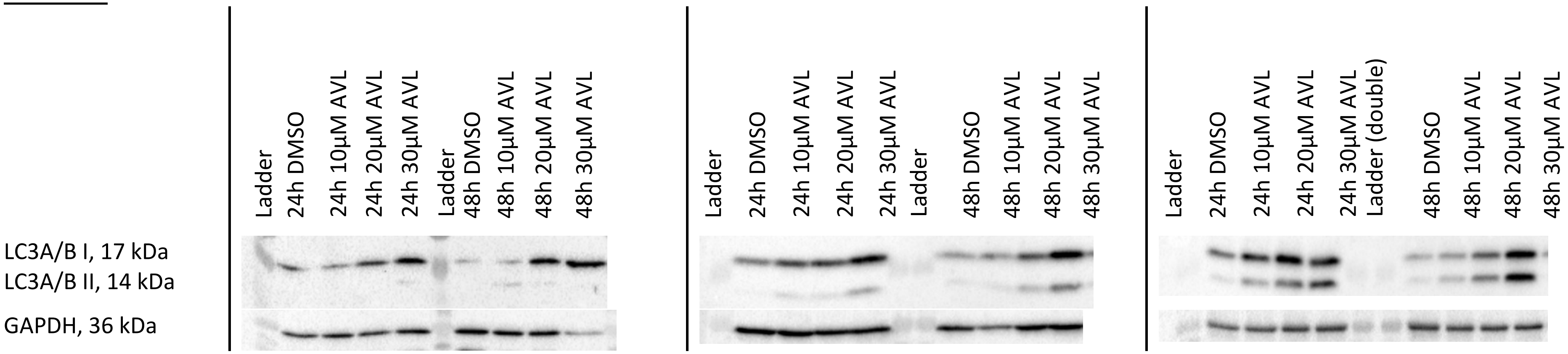

Figure S4: Original membranes of immunoblots shown in Figure 3.

Original blots of Figure 5A

<table>
<tr>
<td>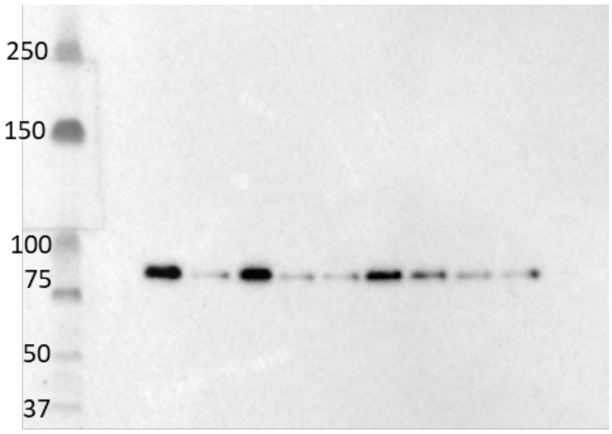
</td>
<td>BTK 7F12H4
(80 kDa)</td>
<td rowspan="2">Lane 1: Marker
Lane 2: Scr 1
Lane 3: BTK 1
Lane 4: Scr OZ 1
Lane 5: BTK OZ 1
Lane 6: BTK PB 1
Lane 7: Scr OZ 5
Lane 8: BTK OZ 5
Lane 9: Scr PB 6
Lane 10: BTK PB 6</td>
</tr>
<tr>
<td>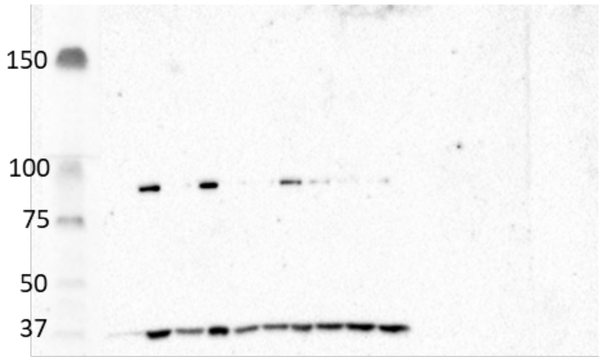
</td>
<td>GAPDH
(37 kDa)</td>
</tr>
</table>

Figure S5: Original membranes of immunoblots shown in Figure 5A.

Original blots of Figure S1A

<table>
<tr>
<td>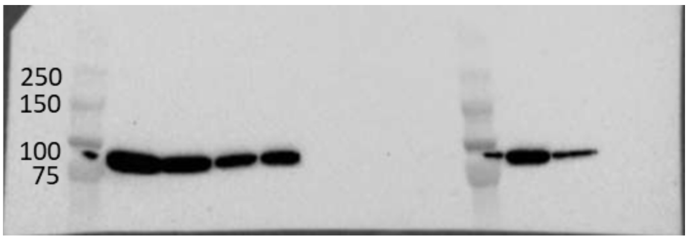
</td>
<td>BTK 7F12H4
(80 kDa)</td>
<td rowspan="2">Lane 1: Marker
Lane 2: Namalwa
Lane 3: UDSCC1
Lane 4: UDSCC5
Lane 5: UDSCC6
Lane 6: Moe1b
Lane 7: Moe1a
Lane 8: TIGKs
Lane 9: marker
Lane 10: not of interest
Lane 11: not of interest</td>
</tr>
<tr>
<td>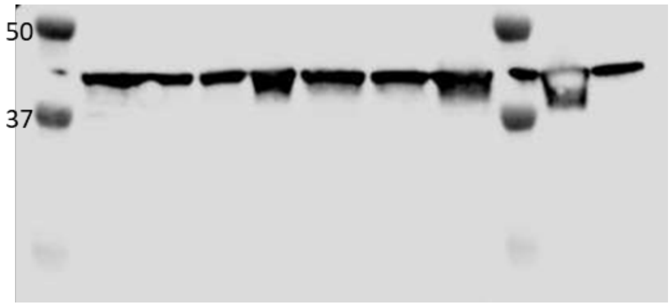
</td>
<td>β-actin
(42 kDa)</td>
</tr>
</table>

Original blots of Figure S1B

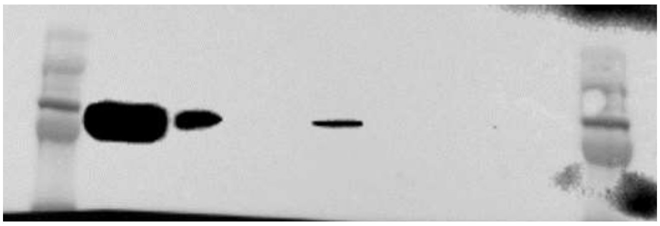	**BTK 7F12H4**<br>(80 kDa)	Lane 1: Marker<br>Lane 2: Namalwa<br>Lane 3: tumor tissue 1<br>Lane 4: tumor tissue 3<br>Lane 5: tumor tissue 2<br>Lane 6: healthy tissue 1<br>Lane 7: healthy tissue 3<br>Lane 8: healthy tissue 2<br>Lane 9: marker
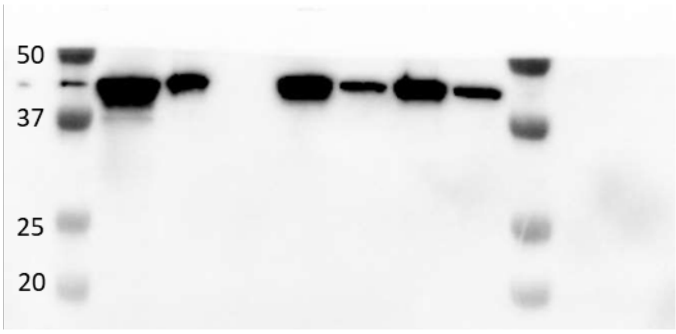	**β-actin**<br>(42 kDa)	

<u>Figure S6:</u> Original membranes of immunoblots shown in Figure S1.

Table S1: *HNSCC cell lines*

Cell line	Sex	Site of origin	TNM stage	HPV 16 status	TP53 gene mutation
**UDSCC1**	M	Oropharynx	T3N2bM0	-	Skip exon 3
**UDSCC2**	M	Hypopharynx	T1N2M0	+	wt
**UDSCC3**	M	Larynx	T2N2cM0	-	Nonsense, p.224Glu->Stop
**UDSCC5**	M	Larynx	T1N1M0	-	Missense, p.179His->Tyr
**UDSCC6**	M	Oropharynx	T2N0M0	-	Missense, p.220Tyr->Cys
**UDSCC8**	/	Larynx	/	-	Missense, p.155Thr->Asn
**UMSCC10A**	M	Larynx	T3N0M0	-	Missense, p.245Gly->Cys
**UMSCC11B**	M	Larynx	T2N2aM0	-	Missense, p.242Cys->Ser
**UMSCC17B**	F	Larynx	T1N0M0	-	wt
**UMSCC22B**	F	Hypopharynx	T2N1M0	-	Missense, p.220Tyr->Cys

Table S2: *Non-tumor oral epithelial cell lines*

Cell line	Sex	Site of origin	Reference
**Moe1a**	unspecified	Oral epithelium	[27]
**Moe1b**	unspecified	Oral epithelium	[27]
**hTERT TIGKs**	M	Gingiva; Epithelium	[28]

Table S3: *Clinical data of patient samples*

Patient	Sex	Site of origin	TNM stage	HPV status
**1**	M	Oral cavity	T2N0M0	n.a.
**2**	M	Oral cavity	T2N0M0	n.a.
